# Frequency of Oral Behaviors as a Risk Factor for Somatosensory Tinnitus

**DOI:** 10.1055/s-0045-1802577

**Published:** 2025-04-15

**Authors:** Ana Carolina de Oliveira Garcia D'Amato, Thaís Spisila, Gabriela Schumacher de Camargo, Priscila Brenner Hilgenberg-Sydney

**Affiliations:** 1 Temporomandibular Disorders and Orofacial Pain Outpatient Service, Department of Ophthalmology-Otorhinolaryngology, School of Medicine, Universidade Federal do Paraná, Curitiba, PR, Brazil

**Keywords:** temporomandibular joint disorders, tinnitus, bruxism, teeth grinding disorders, checklist

## Abstract

**Introduction** Somatosensory tinnitus is a type of tinnitus that can be modified by somatosensory stimuli from the cervical spine and temporomandibular area. Temporomandibular disorders and oral behaviors are associated with a higher prevalence of somatosensory tinnitus, and this association is described in the literature as part of the diagnosis of this condition.

**Objective** To verify the association between somatosensory tinnitus and oral behaviors.

**Methods** Patients were recruited from an Orofacial Pain outpatient clinic and from Head and Neck Unit. All participants underwent anamnesis, physical examination and completed the Oral Behaviors Checklist questionnaire. Forty-six patients were divided into 2 groups, each consisting of 23 patients: somatosensory tinnitus group (STG) and a comparison group (CG), with subjective tinnitus. Data were gathered and analyzed using the Jamovi software (open source) and a significance level of 5% was adopted. Somatosensory tinnitus was associated with dizziness and neck and temporomandibular joint pain.

**Results** There was an association between a higher Oral Behaviors Checklist score and the presence of somatosensory tinnitus. For each point marked on Oral Behaviors Checklist, there was an 8.2% greater chance of having somatosensory tinnitus. Tinnitus modulation through somatic maneuvers and palpation of masticatory and cervical muscles was significantly associated with somatosensory tinnitus.

**Conclusion** Dizziness and neck and temporomandibular joint pain are associated with the presence of somatosensory tinnitus. Probable sleep and awake bruxism are not exclusive behaviors of somatosensory tinnitus patients. However, their frequency may impact its presence.

## Introduction

Tinnitus is an auditory illusion, serving as the symptom of a specific disease and affecting around 10 to 15% of the world population. 1 Its etiology is multifactorial, not yet completely elucidated, with a multitude of potential influencing factors 2 3 4 including systemic causes (diabetes, obesity, inflammatory diseases), functional somatic syndromes, psychiatric disorders and hearing loss, normally with more than one cause present in the same individual. 5 The somatosensory system can cause tinnitus and/or change the tone or volume of an existing tinnitus through somatosensory stimuli from the cervical spine and temporomandibular area. 6 7 8 When somatosensory influence is a major influencing factor, it is termed somatosensory tinnitus (ST), being present in 12 to 43% of patients with tinnitus. 3 9

The modulation of ST is characterized as the alteration of the perception of this sound, occurring through muscular maneuvers, interaction with the temporomandibular joints and movement of the head and neck. Common reasons for activation of somatosensory input are the presence of temporomandibular disorders (TMDs) and/or oral behaviors, such as parafunctions, including awake and sleep bruxism, excessive gum chewing, lip or nail biting), 3 which are also considered etiological factors for TMDs. 10 Up to 85% of patients with ST also have TMDs. 11 12

It is suggested that TMDs and ST are related, as tinnitus occurs in up to 64% of patients with TMDs. 13 Therefore, TMDs are one of the main risk factors for the emergence of ST. 12 In addition to TMDs, another factor that is associated with ST modulation is the presence of bruxism. 14 Previous studies indicate that, especially in patients with ST related to the temporomandibular area, the prevalence of bruxism is very high. 9 15 Just the presence of bruxism, whether awake or during sleep, cannot be used as a diagnostic criterion for tinnitus. However, together with other aspects, especially with modulation in the face of head or neck pain, it is a strong indicator that the tinnitus may have a somatosensory influence. 14 In cases of ST, when TMDs and/or oral behaviors are controlled, tinnitus usually improves, with complete remission or decrease in intensity and frequency of the perceived sound. 12 Although there is some relationship in the literature between these symptoms and ST, it has not yet been completely elucidated. The aim of the present study was to verify the association between ST and oral behaviors.

## Methods

The present study is a cross-sectional observational study. Participants were recruited voluntarily through posters scattered around the Temporomandibular Disorders and Orofacial Pain Outpatient Service, Department of Ophthalmology-Otorhinolaryngology, School of Medicine, Universidade Federal do Paraná, and posted on social media.

The sample size calculation resulted in 23 patients in each group, based on a previous pilot study with patients with somatosensory tinnitus that applied the Oral Behaviors Checklist (OBC) questionnaire, standard deviation (SD) of ± 10.41, with a minimum difference detected of 9 points, considering a 5% alpha error and a 20% beta error. All patients were assessed through anamnesis and a self-administered questionnaire, the OBC and diagnosis of temporomandibular disorder was accessed through the Diagnostic Criteria for Temporomandibular Disorders (DC/TMD). 16

A physical and clinical examination was conducted for each patient, including palpation of the masticatory (masseter, temporalis) and cervical muscles (sternocleidomastoid, trapezius, and suboccipital). All participants were evaluated according to the DC/TMD. 17 Somatic maneuvers, such as turning the head upward, downward, and to the sides, were also performed. These palpations and maneuvers were carried out to evaluate if there was somatic modulation of tinnitus. 3 Somatosensory tinnitus was assessed according to Michiels et al. (2018). 3 18 Probable awake bruxism (PAB) was assessed using the criteria proposed by Lobbezoo et al. (2018). 17

The participants were divided in two groups: the ST group was composed of 23 patients with somatosensory tinnitus, and the comparison group (CG) was composed of 23 patients with subjective tinnitus, without any somatic influence.

Individuals with systemic conditions such as rheumatoid arthritis, hypothyroidism, diabetes, uncontrolled hypertension, leprosy, and/or previously diagnosed disabling psychological and neurological disorders, patients with removable conventional complete dentures in one or both arches, patients with Meniere's disease, people with special needs, and pregnant women were not included. Individuals who did not agree and/or sign or withdraw the free and informed consent form were excluded from the sample.

The results were gathered and evaluated using Jamovi software, version 2.3.21 (open source). A significant level of 5% was adopted for all tests. The t-student test was used for comparisons between the average age and the OBC questionnaire results between groups. The Chi-squared (χ ^2^ ) test was employed for comparisons of the presence of PAB between the groups. A simple regression analysis was performed to evaluate the influence of OBC score on the presence of ST.

## Result

The total sample comprised 46 tinnitus patients, divided into 2 groups. Of these, 58.7% were female, and 41.3% were male. There were no differences between the groups regarding sex ( *p* = 0.765). Table 1 displays the comparisons for mean age, OBC score, and tinnitus intensity between groups. The simple logistic regression analysis revealed that for each additional point on the OBC questionnaire, the odds of having ST increase by 8.2% (χ ^2^ = 9.98; *p* = 0.002; R ^2^_N_ = 0.260), with an odds ratio (95% confidence interval [95%CI]) of 1.082 (1.021–1.145).

**Table 1:** Mean age, oral behaviors checklist score, and tinnitus intensity between groups

	ST group	Comparison group	*p* -value
Age	35.9(± 10.6)	34.4(± 14.3)	0.684
OBC	33.3(± 12.9)	20.9(± 13.1)	0.002*
Tinnitus intensity	5.96(± 2.60)	5.43(± 2.09)	0.457

**Abbreviations:** OBC, oral behaviors checklist; ST, somatosensory tinnitus.

**Note:** *Statistically significant.

There were no significant differences between the two groups regarding sex, tinnitus time of onset ( *p* = 0.814), location ( *p* = 0.611), worst side ( *p* = 0.340), quality ( *p* > 0.05), volume fluctuation ( *p* = 0.353), evolution ( *p* = 0.502), frequency ( *p* = 0.767), and noise effect ( *p* = 0.298). Also, there was no statistical difference between groups regarding the presence of probable awake or sleep bruxism, otalgia ( *p* = 0.114), headache report ( *p* = 0.491), hyperacusis ( *p* = 0.552), and hypoacusis ( *p* = 0.767). However, a significant association was found between ST and dizziness ( *p* = 0.018), neck pain ( *p* = 0.007) and temporomandibular joint (TMJ) pain ( *p* = 0.013) as shown in Table 2 .

**Table 2:** Presence of dizziness, neck pain, and temporomandibular joint pain among groups

	ST group	Comparison group	*p* -value
Dizziness	15 (65.2%)	7 (30.4%)	0.018*
Neck pain	18 (78.3%)	9 (39.1%)	0.007*
TMJ pain	19 (82.6%)	10 (43.5%)	0.013*

**Abbreviations:** ST, somatosensory tinnitus; TMJ, temporomandibular joint.

**Note:** *Statistically significant.

All somatic maneuvers and palpation of masticatory and cervical muscles were positively associated with the ST group ( Table 3 ).

**Table 3:** Presence of trigger points and modulation maneuvers characteristics

	ST group	Comparison group	*p* -value
*Affirmations for somatosensory tinnitus diagnosis*			
Simultaneous onset of tinnitus and neck/jaw pain	8 (34.8%)	0 (0.00%)	0.001*
Influence of posture	13 (56.5%)	0 (0.00%)	< 0.001*
Covariation of tinnitus and neck/jaw pain	11 (47.8%)	0 (0.00%)	0.0001*
*Trigger points*			
Masseter muscle	12 (52.17%)	2 (8.70%)	0.001*
Temporalis muscle	12 (52.17%)	3 (13.04%)	0.004*
Sternocleidomastoid muscle	9 (39.13%)	0 (0.00%)	0.0008*
Trapezius muscle	9 (39.13%)	0 (0.00%)	0.0008*
Suboccipital muscles	8 (34.87%)	0 (0.00%)	0.001*
*Modulation maneuvers*			
Dental clenching	16 (69.57%)	6 (26.09%)	0.003*
Open your mouth with resistance	16 (69.57%)	3 (13.04%)	< 0.001*
Protrude jaw with resistance	17 (73.91%)	3 (13.04%)	< 0.001*
Laterality with resistance	16 (69.57%)	5 (21.74%)	0.001*
Head up, to the right, to the left, forward, and backward	10 (43.48%)	1 (4.35%)	0.001*
Rotate the head to the left against resistance in the ipsilateral zygomatic arch	9 (39.13%)	2 (8.70%)	0.015*
Rotate the head to the right against resistance in the ipsilateral zygomatic arch	9 (39.13%)	2 (8.70%)	0.015*
Rotate the head to the left and tilt to the right with resistance applied to the right temple.	8 (34.87%)	1 (4.35%)	0.009*
Rotate the head to the right and tilt to the right with resistance applied to the left temple	8 (34.87%)	0 (0.00%)	0.001*

**Abbreviation:** ST, somatosensory tinnitus.

**Note:** *Statistically significant.

## Discussion

This cross-sectional observational study found that the OBC score has an impact on the presence of ST. It is already recognized that bruxism may play a role in the diagnostic of ST, as stated by Michiels et al. (2022). 14 According to the results of the present study, a higher frequency of oral behaviors may be a risk factor for the development of ST.

There was no difference between the gender and age of the participants in each of the studied groups. Tinnitus may present in both males and females. Somatosensory tinnitus is common among TMD patients, 11 typically with a mean age of 25 to 45 years, aligning with the findings of the present study.

This cross-sectional observational study found that the OBC score has an impact on the presence of ST. The odds of having ST increase by 8.2% for each point in OBC score. The association between ST and oral behaviors has already been explored in the study of Cimino et al. (2022), 19 who verified the higher the score on the OBC questionnaire, the greater the severity of subjective tinnitus. Michiels et al. (2018) 3 also highlights the important role of oral behaviors on the presence of ST.

Despite that, the study claims that it is not possible to state that there is a causal relationship between the frequency of oral behaviors and the severity of tinnitus. However, due to this significant positive correlation in other studies, it is suggested that the abnormal activity of masticatory muscle and TMJ may induce a more intense tinnitus feeling. 12 20 21 22

There was a significant association between ST and dizziness ( *p* = 0.018). Although this association is not consolidated in the literature, a recent study showed that patients with ear fullness, dizziness, and female sex are more likely to present tinnitus. It is noted that patients with dizziness show greater pain intensity, and in patients experiencing TMD and dizziness simultaneously, the pain will be more intense than in patients without dizziness. 23 As a result of our study, we also found that there was a significant association between ST and neck pain and TMJ pain, which is in agreement with the results demonstrated in previous studies. 12 20 24 25 It is important to emphasize that although there is a proven association between tinnitus and frequent pain in the head, neck, or tinnitus accompanied by TMD, these conditions can occur simultaneously in a patient without a causal relationship between them. 3 26

The tinnitus modulation by voluntary movements or somatic maneuvers of head and neck, pressure in myofascial trigger points, bad posture, painful TMD and neck pain are part of the diagnosis of ST. 3 12 21 22 27 In the present study, all somatic maneuvers and palpation of masticatory and cervical muscles were positively associated with the ST group. However, somatic modulation with muscles palpation and/or somatic maneuvers are not an exclusive characteristic of somatosensory tinnitus. Somatic modulation may also occur in individuals with other subtypes of tinnitus, in accordance with previous authors. 3

Regarding bruxism, the present study presents different information from what is found in the literature. Bruxism, both sleep and awake, did not show specific association with ST, contrary to what other studies 12 27 indicate, including when using bruxism as a means for differential diagnosis of ST. 3 Therefore, bruxism should not be used as the sole criterion to the diagnosis of ST, but rather as part of holistic patient evaluation. Other criteria, such as head and neck pain simultaneous with tinnitus modulation and tension on the suboccipital muscle, hold more value for the diagnosis. 14

This lack of association between awake and sleep bruxism with ST may be related to the recruitment of this research sample. Patients were screened at dental clinics that treat these conditions, which would explain the prevalence of probable bruxism in around 93% of participants. The use of ecological momentary assessment of bruxism could also enhance the determination of awake bruxism frequency and contribute to diagnosing and understanding its influence on the presence of ST. Another limitation of the present study was the absence of a standardized audiological evaluation as an inclusion criterion. However, according to some authors, there appears to be a connection between somatic disorders and the typical hearing level in patients experiencing tinnitus. Additionally, somatosensory effects may manifest in individuals with hearing impairment. 12 28

## Conclusion

The present study indicates that probable sleep and awake bruxism are not exclusive behaviors of ST patients. However, their frequency may influence the presence of ST. Additionally, dizziness, neck and TMJ pain are associated with the presence of ST.
